# A Mild Case of Autosomal Recessive Osteopetrosis Masquerading as the Dominant Form Involving Homozygous Deep Intronic Variations in the *CLCN7* Gene

**DOI:** 10.1007/s00223-022-00988-8

**Published:** 2022-05-26

**Authors:** Jochen G. Hofstaetter, Gerald J. Atkins, Hajime Kato, Masakazu Kogawa, Stéphane Blouin, Barbara M. Misof, Paul Roschger, Andreas Evdokiou, Dongqing Yang, Lucian B. Solomon, David M. Findlay, Nobuaki Ito

**Affiliations:** 1 1st Medical Dept., Hanusch Hospital, Ludwig Boltzmann Institute of Osteology at Hanusch Hospital of OEGK and AUVA Trauma Centre Meidling, Vienna, Austria; 2 Michael Ogon Laboratory, Orthopaedic Hospital Vienna-Speising, Vienna, Austria; 3 Centre for Orthopaedic & Trauma Research, Faculty of Health and Medical Sciences, Adelaide Health and Medical Sciences Building, The University of Adelaide, North Terrace, Adelaide, SA 5005 Australia; 4 Division of Nephrology and Endocrinology, The University of Tokyo Hospital, Tokyo, Japan; 5 Osteoporosis Center, The University of Tokyo Hospital, Tokyo, Japan; 6 Department of Orthopaedics and Trauma, Royal Adelaide Hospital, Adelaide, SA 5000 Australia

**Keywords:** ADOII, ARO, Bone mineralization density distribution, CLCN7, Osteocyte lacunae, Cement lines

## Abstract

Osteopetrosis is a heterogeneous group of rare hereditary diseases characterized by increased bone mass of poor quality. Autosomal-dominant osteopetrosis type II (ADOII) is most often caused by mutation of the *CLCN7* gene leading to impaired bone resorption. Autosomal recessive osteopetrosis (ARO) is a more severe form and is frequently accompanied by additional morbidities. We report an adult male presenting with classical clinical and radiological features of ADOII. Genetic analyses showed no amino-acid-converting mutation in *CLCN7* but an apparent haploinsufficiency and suppression of *CLCN7* mRNA levels in peripheral blood mononuclear cells. Next generation sequencing revealed low-frequency intronic homozygous variations in *CLCN7*, suggesting recessive inheritance. In silico analysis of an intronic duplication c.595-120_595-86dup revealed additional binding sites for Serine- and Arginine-rich Splicing Factors (SRSF), which is predicted to impair *CLCN7* expression. Quantitative backscattered electron imaging and histomorphometric analyses revealed bone tissue and material abnormalities. Giant osteoclasts were present and additionally to lamellar bone, and abundant woven bone and mineralized cartilage were observed, together with increased frequency and thickness of cement lines. Bone mineralization density distribution (BMDD) analysis revealed markedly increased average mineral content of the dense bone (CaMean *T*-score + 10.1) and frequency of bone with highest mineral content (CaHigh *T*-score + 19.6), suggesting continued mineral accumulation and lack of bone remodelling. Osteocyte lacunae sections (OLS) characteristics were unremarkable except for an unusually circular shape. Together, our findings suggest that the reduced expression of *CLCN7* mRNA in osteoclasts, and possibly also osteocytes, causes poorly remodelled bone with abnormal bone matrix with high mineral content. This together with the lack of adequate bone repair mechanisms makes the material brittle and prone to fracture. While the skeletal phenotype and medical history were suggestive of ADOII, genetic analysis revealed that this is a possible mild case of ARO due to deep intronic mutation.

**Supplementary Information:**

The online version contains supplementary material available at 10.1007/s00223-022-00988-8.

## Introduction

Osteopetrosis is a group of rare heterogeneous hereditary diseases characterized by increased bone mass, which is caused by reduced bone resorption, due to reduced numbers and or impaired function of osteoclasts [1]. Autosomal-dominant osteopetrosis type II (ADOII) (MIM# 166600), also known as Albers-Schönberg disease, is the most frequent (1:20.000) form of osteopetrosis [2] and is mostly caused by heterozygous-dominant negative mutations of the gene-encoding chloride channel, voltage-sensitive 7 (*CLCN7*; OMIM #602727) [3–5]. The autosomal recessive form (ARO) (MIM# 611490) is less frequent [4, 6]. The protein product of *CLCN7* has an important role in mature osteoclasts, extruding chloride from the ruffled boarder to facilitate proton extrusion by V-ATPase [5–7], thus, providing the ability to acidify the extracellular environment. Interestingly, osteopetrotic conditions based on *CLCN7* mutations show great variation in their presentation and severity [3].

ADOII individuals attain average height and have normal life expectancy, but X-rays reveal high bone mass and characteristic “sandwich vertebrae” [1]. Despite the dramatic increase in dual-energy X-ray Absorptiometry (DXA)-derived areal bone mineral density (BMD), ADOII individuals have a high risk of sustaining low energy fractures. ARO, on the other hand, is a severe form presenting in early childhood, and while it shares many clinical skeletal features of ADOII, is also characterized by morbidities, such as cranial neuropathy, including palsy, blindness, and deafness, and is often fatal in childhood [8].

We report here a case of a middle-aged male of normal height, who at surgery for a subtrochanteric fragility fracture of the femur was found to have unusually high bone mass. ADOII was suspected based on clinical history and radiographical features. Consequently, next-generation sequencing analysis was performed to identify contributing genetic changes, and a transiliac bone biopsy sample was taken for examinations of histological and material abnormalities using light microscopy and scanning electron microscopy. In particular, we have successfully used quantitative backscattered electron imaging (qBEI) for the characterization of the bone mineralization phenotype in patients with genetic diseases, thus, providing insights into the genotype-bone phenotype relationship [9–11].

## Materials and Methods

Clinical history taking, physical examination and plain X-ray were performed at the Royal Adelaide Hospital during the patient’s admission for right proximal femur fracture. Research ethics approval for the study was obtained from the Royal Adelaide Hospital Human Research Ethics Committee (Approval No. RAH130114) and informed consent to both conduct the study and publish the findings was obtained from the proband.

### Clinical Description of Patient

**Fig. 1:**
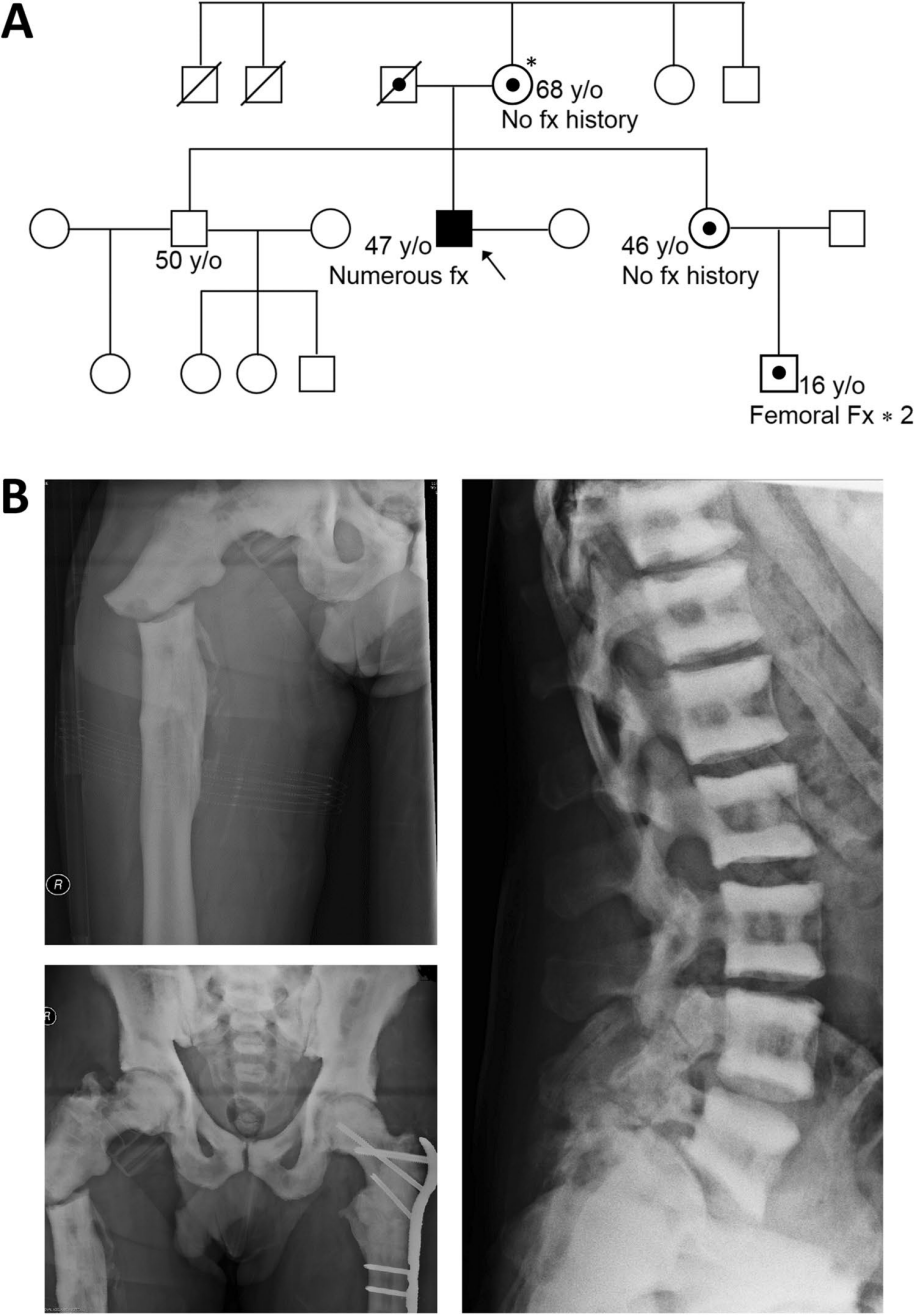
**A** The proband’s pedigree, showing mother, sister and nephew, who are apparently heterozygous for the same genetic abnormalities. **B** Radiographs of the proband, showing the right proximal femoral fracture, the previously treated left femoral fracture, the extremely radio-dense skeleton and the “sandwich” vertebrae, typical of ADOII. *Heterozygosity/carrier status of the proband’s relatives was assumed based on available radiographs and clinical histories, although this was not confirmed by genetic analysis

The proband was the second child of presumed non-consanguineous parents. Several of the proband’s relatives, his mother, younger sister and his nephew, had at least some features of ADOII including radiographic hallmarks of typical “sandwich” vertebrae. However, only his nephew had any a history of fracture, having incurred a tibial fracture on two occasions during exercise (Fig. 1A).

The proband reported no problems with dentition. He is an occasional smoker with a history of moderate alcohol consumption. In contrast to the unremarkable fracture histories of his relatives, at 5 years of age, he suffered his first fracture in his right olecranon after falling off a low fence and was clinically diagnosed with ADOII/Albers-Schönberg disease from his distinctive X-ray images. He suffered at least 30 further fractures throughout his childhood and adulthood at multiple skeletal sites, mainly from relatively low impact traumas. Between ages 46 and 47, he suffered fractures to his left proximal femur, which fractured again after several months and then his right proximal femur also fractured (Fig. 1B). Plain radiographs revealed a remarkably high bone mass throughout his body, with “sandwich” or endobone vertebrae (Fig. 1B). Blood tests showed no evidence of anaemia, leukocytopaenia or other abnormalities. He underwent surgery where it was found that the femur could not be nailed because of insufficient endosteal space and that the hardness of the cortical bone made drilling screw holes and plating difficult. A bone biopsy sample was obtained from the right iliac crest at the time of surgery for examination of histological and bone material quality aspects. Furthermore, genetic analysis from a blood sample and an osteoclastogenesis assay were performed.

### Sequencing

Genomic DNA (gDNA) and total RNA were isolated from peripheral blood mononuclear cells (PBMC). Total RNA and complementary DNA (cDNA) were prepared, as described previously [12]. gDNA and complementary DNA (cDNA) sequencing for *CLCN7,* including splice sites and 5′, 3′ untranslated regions (5′, 3′ UTR) were sequenced using Applied Biosystems 3730 and 3730xl sequencers (Applied Biosystems, Carlsbad, CA, USA). Whole Genome Sequencing was performed (Illumina NovaSeq 6000, Australian Genome Research Foundation, Melbourne, VIC, Australia). All amino-acid changing variants in the 16 genes responsible for the development of osteopetrosis or high bone mass were extracted irrelevant to the frequency, while only low-frequency variants (< 0.5% in the general population by gnomAD [13] and 1000 Genomes [14]) were explored in the intronic regions of *CLCN7*. Quantitative reverse transcription (RT)-PCR was performed for *CLCN7* and glyceraldehyde-3-phosphate dehydrogenase (*GAPDH*) mRNA, using RNA extracted from PBMC of the proband and 6 healthy controls, as described previously [12]. Methylation-specific PCR for the *CLCN7* gene was also conducted, as described previously [15]. In silico analysis to detect Serine and Arginine-rich Splicing Factor (SRSF)-binding sites in the introns of *CLCN7* was conducted using ESEfinder3.0 [16].

### Osteoclastogenesis Assay

Peripheral blood mononuclear cells (PBMC; 5.4 × 10^5^ cells/cm^2^) were incubated in αMEM medium containing added recombinant human (rh)M-CSF (25 ng/ml) and rhRANKL (100 ng/ml). Media were replaced every 3 days thereafter. Osteoclast number was assessed by TRAP staining after 6–9 days, as described previously [17].

### Iliac Crest Bone Biopsy

#### Sample Preparation

A routine Bordier transiliac biopsy could not be performed due to the increased hardness of the patient’s bone material. Instead, a diamond drill was used to drill two small holes into the iliac crest, and subsequently, a bone chip could be obtained from the iliac crest. This bone sample was immediately fixed in 70% ethanol, dehydrated in a graded series of ethanol and embedded undecalcified in polymethylmethacrylate. Subsequently, 3 µm sections for histology/histomorphometry were prepared with a hard tissue microtome (Leica SM2500, Nussloch, Germany). The surface of the residual sample block was prepared by grinding and polishing (Logitech PM5, Glasgow, Scotland) and carbon coated (Agar SEM Carbon Coater; Agar Scientific, Stansted, UK) for quantitative backscattered electron imaging (qBEI) evaluation.

#### Histological Assessment

As the bone biopsy sample showed predominantly mineralized tissue and contained almost no free bone marrow space, we could not quantify standard histomorphometric parameters. However, analysis of qBEI overview images of the sample allowed us to determine mineralized tissue volume per tissue volume (corresponding to bone volume per tissue volume, BV/TV). The Goldner’s trichrome or Giemsa-stained bone sections were imaged in a light microscope (Axiophot, equipped with digital camera AxioCam HRc, Zeiss, Oberkochen, Germany). The histologic sections were used to assess the presence and appearance of osteoclasts and to discriminate areas of lamellar bone and woven bone using polarized light microscopy. The presence of residual mineralized cartilaginous matrix could be quantified by qBEI based on its non-fibrillar micro-texture and higher mineral content. Cement line phenotyping as well as osteocyte lacunae characteristics were also performed on basis of qBEI (following).

#### Quantitative Backscattered Electron Imaging (qBEI)

qBEI analysis of bone tissue is based on the fact that the grey levels in the images (if adequately calibrated) are proportional to the local weight percentage of calcium (Ca) in the bone matrix as described elsewhere [18]. We acquired qBEI images in Zeiss DSM 962 and Zeiss Supra 40 instruments (both Zeiss, Oberkochen, Germany) for quantification of global and local calcium content within the biopsy sample, including the bone mineralization density distribution (BMDD), the BV/TV, tissue details like mineralized cartilage, cement lines and osteocyte lacunae sections (OLS). Both instruments were equipped with a four-quadrant semiconductor backscatter electron detector and operated at 20 kV accelerating voltage for the beam electrons and a scan speed of 100 s per frame.

In order to determine the patient’s BMDD, the Zeiss DSM 962 was operated at 110 pA probe current and 15 mm working distance. A series of images with a pixel resolution of 3.6 μm from about 19 mm^2^ of the iliac crest bone area were recorded. Grey-level histograms were derived from the qBEI images representing frequency distributions of pixels with a certain Ca content (Ca weight%), denominated BMDD. Five parameters were deduced from this BMDD: The weighted mean Ca concentration of the bone area (Ca_Mean_), the peak position of the histogram (Ca_Peak_, indicating the most frequent Ca concentration), the full width at half maximum of the distribution (Ca_Width_, indicative for the heterogeneity in matrix mineralization), the percentage of bone areas having a Ca concentration lower than 17.68 wt% Ca (Ca_Low_), and the percentage of bone areas having a Ca concentration higher than 25.30 wt% Ca (Ca_High_, corresponding to fully mineralized bone areas, mainly interstitial bone and cement lines). These parameters were compared to reference values for cancellous BMDD for adult individuals [19].

For the assessment of BV/TV, a binary image (discriminating between bone marrow and mineralized matrix) was generated from the entire sectioned bone area. Discrimination between bone marrow space and bone area was performed by a at fixed grey-level threshold corresponding to 0.87 weight% Ca. Image analysis and quantification of the two areas were performed by custom-made routines using the software ImageJ [20].

In order to study mineralized tissue details, the Zeiss Supra 40 operating at about 280 pA probe current, 10 mm working distance and a pixel resolution in the range of 1.76 up to 0.25 µm was used. Additionally, energy dispersive X-ray (EDX) measurements for semi-quantitative information on elemental composition were performed on small areas of mineralized bone, cartilage and cement lines using an EDX system coupled to the SEM SUPRA 40 and equipped with an EDS Silicon Drift detector (X-Max, Oxford Instrument, UK). The energy of the beam electrons was adjusted to 10 keV, and the EDX spectra were analysed based on Oxford INCA software.

The OLS analysis is a 2D characterization of osteocyte lacunae sections based on qBEI images with a pixel resolution of 0.88 μm, which were subsequently transformed to binary images (distinguishing mineralized from unmineralized bone) using a threshold based on a fixed calcium value of 5.2 wt% Ca [21] (mineralized cartilage areas were excluded for OLS analysis). The OLS was extracted in these images using a size range between 5 and 200 µm^2^, respectively, and analysed for the OLS density (the number of OLS per mineralized bone area), the OLS porosity (the total area of OLS given as percentage of the total bone area), the mean OLS area, the mean OLS perimeter, and the mean OLS aspect ratio (the major axis over the minor axis of the fitted ellipse) based on a custom-made macro in ImageJ software. OLS aspect ratio = 1 indicates a circle and increasing values indicate increasingly elongated shape of the OLS. Details of assessment of OLS characteristics are described elsewhere [22]. The presence and viability of the osteocytes within the lacunae cannot be evaluated by this method. The patient’s OLS characteristics were compared with those measured in transiliac bone biopsy samples from two healthy adult women published previously [23].

## Results

### No Amino-acid-converting Mutation in the *CLCN7* Gene

**Fig. 2:**
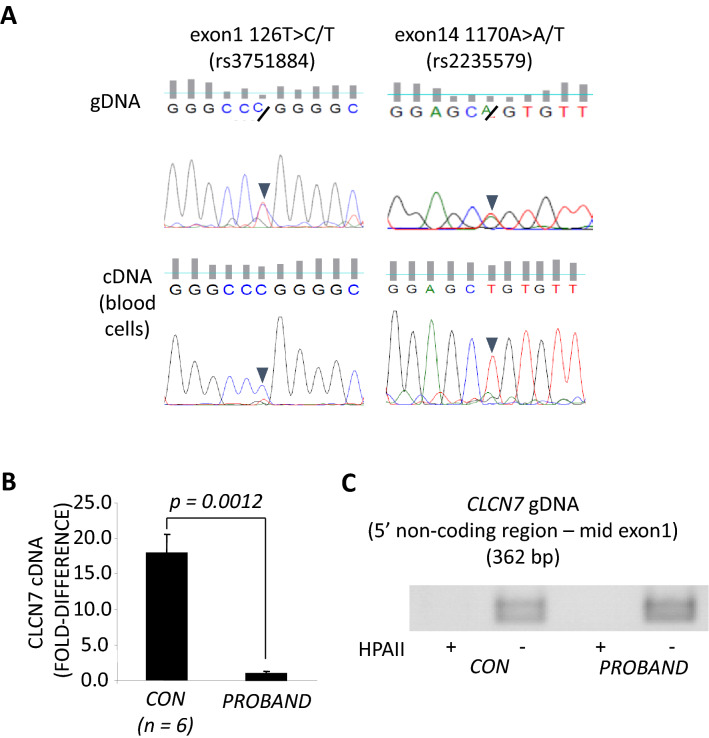
**A** Evidence for two different heterozygous single nucleotide polymorphisms (SNP) were found in exon 1 (c.126T > C/T, rs3751884) and exon 14 (c. 1170A > A/T). **B** Quantitative PCR for *CLCN7* normalized to *GAPDH* expression in PBMC isolated from the proband or 6 healthy controls (CON). **C** gDNA extracted from the proband was completely digested by HPAII, as seen for gDNA from a healthy control

Because mutation of the *CLCN7* chloride channel gene is known to be causative for ADOII, we performed whole gene sequencing of *CLCN7*, using genomic DNA (gDNA) extracted from the proband’s peripheral blood monocytes. We focused on the coding sequence, together with splice sites and predicted 5′ and 3′ UTRs of the *CLCN7* gene. No amino-acid-converting mutations could be found in the proband’s *CLCN7* gene sequence. However, two different heterozygous single nucleotide polymorphisms (SNP) were detected in exon 1 (c.126T > C/T, rs3751884) and exon 14 (c. 1170A > A/T) (Fig. 2A). The presence of these two SNPs (each existing in almost 50% of the population) allowed us to investigate haploinsufficiency of *CLCN7* mRNA expression. Sequencing of exon 1 and exon 14 cDNA revealed that *CLCN7* mRNA is only transcribed from one allele in the proband (Fig. 2A). Furthermore, quantitative PCR for *CLCN7* corrected by *GAPDH* levels showed that the expression level of *CLCN7* mRNA was less than 10% than that of a panel (*n* = 6) of healthy controls (Fig. 2B).

The UCSC Genome Bioinformatics algorithm (http://genome.ucsc.edu/) showed highly conserved CpG islands throughout exon 1 of the *CLCN7* gene and the adjoining non-coding 5′-region. Therefore, to investigate the possibility that promoter methylation was responsible for the haploinsufficiency, methylation-specific semi-quantitative PCR for this site was conducted using the methylation-sensitive restriction enzyme HPAII [15]. However, gDNA extracted from both the proband and a healthy control was completely digested by HPAII (Fig. 2C), suggesting another cause for differential transcription from the *CLCN7* gene in this case.

Whole genome sequencing was then performed to examine exonic sequences of other potentially causative genes, as well as the promoter and deep intronic sequences of *CLCN7* for the presence of low-frequency variants. The sequence data obtained were of high quality, with a median coverage depth of 42.0 (insert size median: 479.0, covered over 10x: 96.74%, 20x: 93.96%, 50x: 18.61%). Exonic sequences were examined for all variants independent of the frequency in all 16 known genes responsible for the development of osteopetrosis, including the recently identified *SLC4A2* [24], and related disorders with high bone mass (*SOST*: sclerosteosis, *CTSK*: pycnodysostosis, *LRP5/6*: high bone mass). The only potentially influencing mutation detected was in *FERMT3*, c.104G > A, p.Arg215Gln, a variation associated with very low frequency (0.00002699) (Table 1).

**Table 1:** Exonic variants in genes responsible for osteopetrosis or high bone mass

Gene	Variant*	Amino acid change	Allele frequency (gnomAD)	In silico prediction	
				SIFT	PolyPhen-2
*CA2*	ND	ND	ND	ND	ND
*CLCN7*	ND	ND	ND	ND	ND
*CTSK*	ND	ND	ND	ND	ND
*FERMT3*	c.104G > A, Het	p.Arg215Gln	0.00002699	Tolerated	Possibly damaging
*IKBKG*	ND	ND	ND	ND	ND
*LRP5*	c.3989C > T, Het	p.Ala1330Val	0.1301	Tolerated	Benign
*LRP6*	c.3184G > A, Het	p.Val1062Ile	0.8492	Tolerated	Benign
*OSTM1*	ND	ND	ND	ND	ND
*PLEKHM1*	ND	ND	ND	ND	ND
*SLC4A2*	ND	ND	ND	ND	ND
*SNX10*	ND	ND	ND	ND	ND
*SOST*	ND	ND	ND	ND	ND
*TCIRG1*	ND	ND	ND	ND	ND
*TMEM53*	ND	ND	ND	ND	ND
*TNFRSF11A*	ND	ND	ND	ND	ND
*TNFSF11*	ND	ND	ND	ND	ND

*ND* not detected; *Het* heterozygous; *gnomAD* the genome aggregation database

*Variants other than synonymous variants are listed

**Fig. 3:**
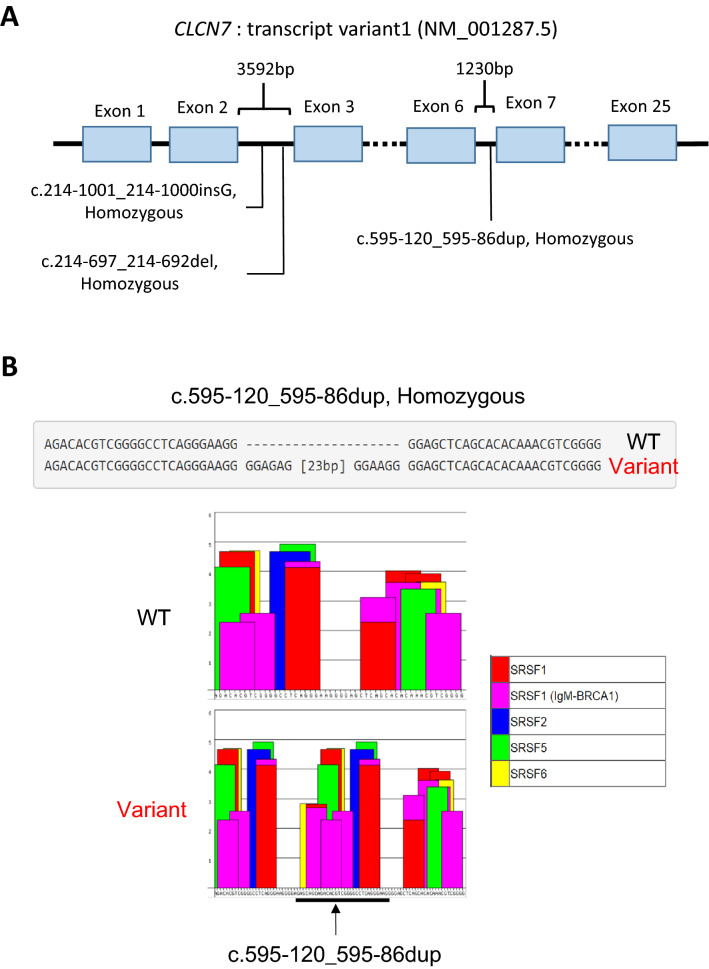
Next generation sequencing analysis of proband *CLCN7* gene. **A** Analysis of intronic low-frequency changes in the *CLCN7* gene. Homozygous sequence duplication (dup), deletion (del) and insertion (ins) are indicated at the respective intronic positions. **B** In silico analysis of the c.595-120_595-86dup insertion for SRSF sites (ESEfinder3.0)

The 2 kb proximal promoter region was free of any low-frequency variant (< 0.5% in the general population). However, some such variants were found in the deep intronic regions, in a homozygous manner (Fig. 3A), suggesting that the proband was a child of a distant consanguineous relationship. These included a relatively large duplication found between exons 6 and 7: c.595-120_595-86dup. This homozygous change may contribute to the low expression of *CLCN7* as in silico analysis revealed this variant potentially renders additional alternate splicing sites for SRSF1, SRSF1 (IgM-BRCA1), SRSF2, SRSF5, and SRSF6 (Fig. 3B). Overall, these findings suggest that ARO is a more accurate diagnosis than ADO II in this case.

### Intact Ability to Differentiate from Monocytes to Osteoclasts

To investigate the likelihood that the proband had a deficiency in osteoclastogenesis, we performed an in vitro analysis. The proband’s PBMC retained the ability to differentiate into large multinucleated TRAP-positive osteoclast-like cells when cultured with exogenous rhRANKL and rhM-CSF (Supplementary Fig. S1), suggesting that osteoclast formation was unimpaired.

### Abnormalities in Phenotype of Bone Histology and Mineralization

#### Histology

**Fig. 4:**
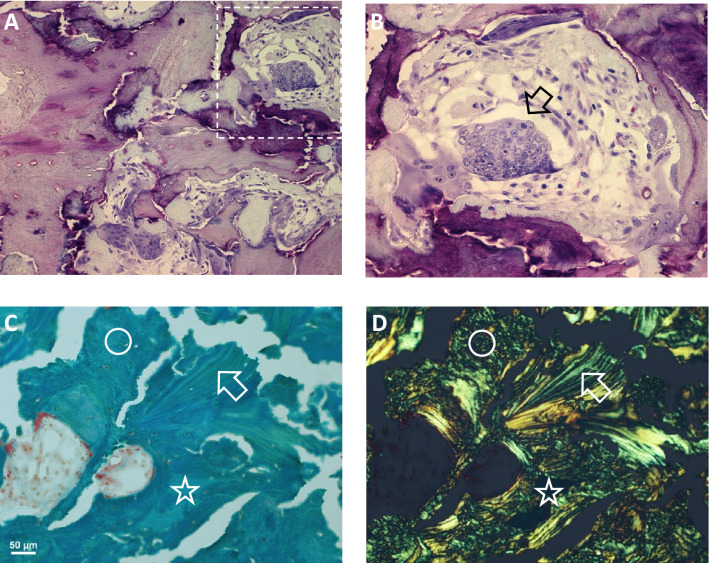
Histological evaluation of the bone biopsy. **A**, **B** Giemsa-stained bone sections under light microscopy. Image in **B** is a detail of **A** (indicated by the dashed area) revealing a giant osteoclast (*arrow*) with a high number of nuclei. **C**, **D** Goldner-stained section under normal light microscopy (**C**) and under polarized light (**D**). The images give evidence for the presence of lamellar bone (*arrow*), woven bone (*circle*) and mineralized cartilage (*asterisk*) as frequently found in the patient’s iliac crest sample

**Fig. 5:**
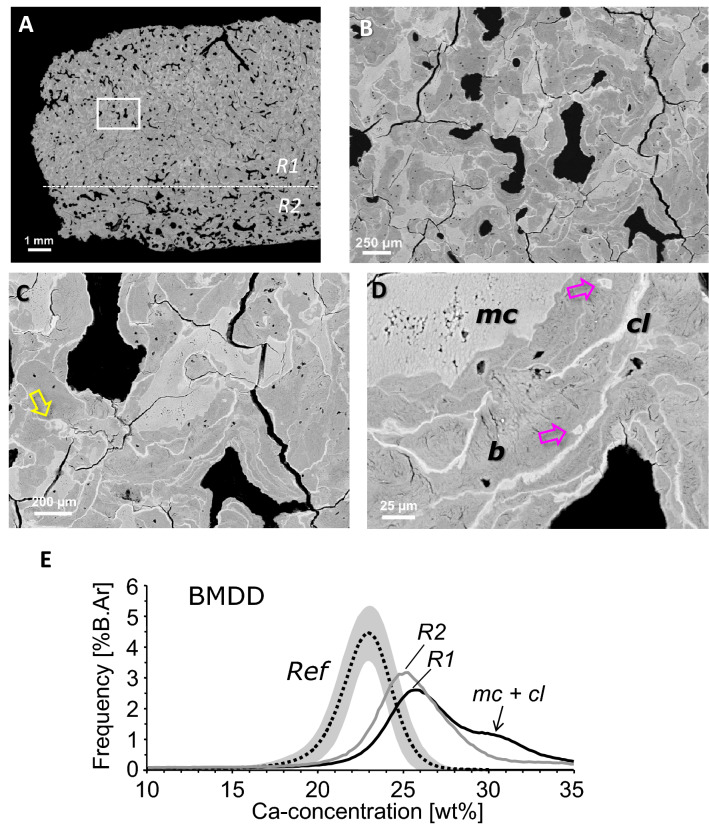
qBEI overview image of the proband’s iliac bone biopsy sample (**A**), showing zones of differential mineral density (shades of grey white). A dense region R1 nearly completely filled with bone matrix, and a less dense region R2 can be distinguished. The corresponding bone mineralization density distribution (BMDD) histograms within these zones are shown in **E**. **B**–**D** Area indicated by the rectangular box at higher magnifications. Clearly visible are the areas of mineralized cartilage (mc) and the cement lines (cl) by their brighter grey levels compared to bone (b), areas indicated in **D**. Of note is the unusual structure of cement lines with small loops (yellow arrow in **C**) and kinks, as well as mineralized osteocyte lacunae (pink arrows in **D**). **E** The BMDD of the proband’s bone at both regions R1 and R2 is clearly shifted to higher calcium concentrations compared to trabecular bone from healthy adults (Reference BMDD, mean and 95th CI indicated by dotted line and grey area, respectively). The “shoulder” (marked by the arrow) reflects the contribution of mineralized cartilage and cement lines to the BMDD

Images of histological staining are shown in Fig. 4. Most remarkable, the transiliac bone sample from our patient did not contain typical trabecular features within the marrow space. The tissue volume was almost completely filled with bone material (Fig. 5A) with BV/TV of 95.5% and 84.6% in regions R1 and R2, respectively.

Despite of the compactness of bone tissue, typical secondary osteons (with concentric bone lamellae) could only rarely be observed. The bone sample contained canals consistent with primary osteons. Giemsa staining gave evidence for the presence of a mixture of bone and cartilage (Fig. 4A). By setting a threshold of 27.9 weight% Ca in the qBEI images (Fig. 5) for demarcation of mineralized cartilage (mineral content beyond threshold) from bone (below threshold), the amount of mineralized cartilage was found to be 32.3% of the mineralized tissue area in region 1 (R1 in Fig. 5A). Consistent with our observation of apparent exuberant osteoclast differentiation from the proband’s PBMC, giemsa-stained sections showed the presence of giant osteoclasts with numerous nuclei (Fig. 4B). Goldner-stained sections examined by polarized light microscopy showed that the bone sample was only partly lamellar (collagenous fibrils arranged into regular lamellae), whereas another part consisted of woven bone (disorganized fibrillar structures) (Fig. 4C, D).

**Fig. 6:**
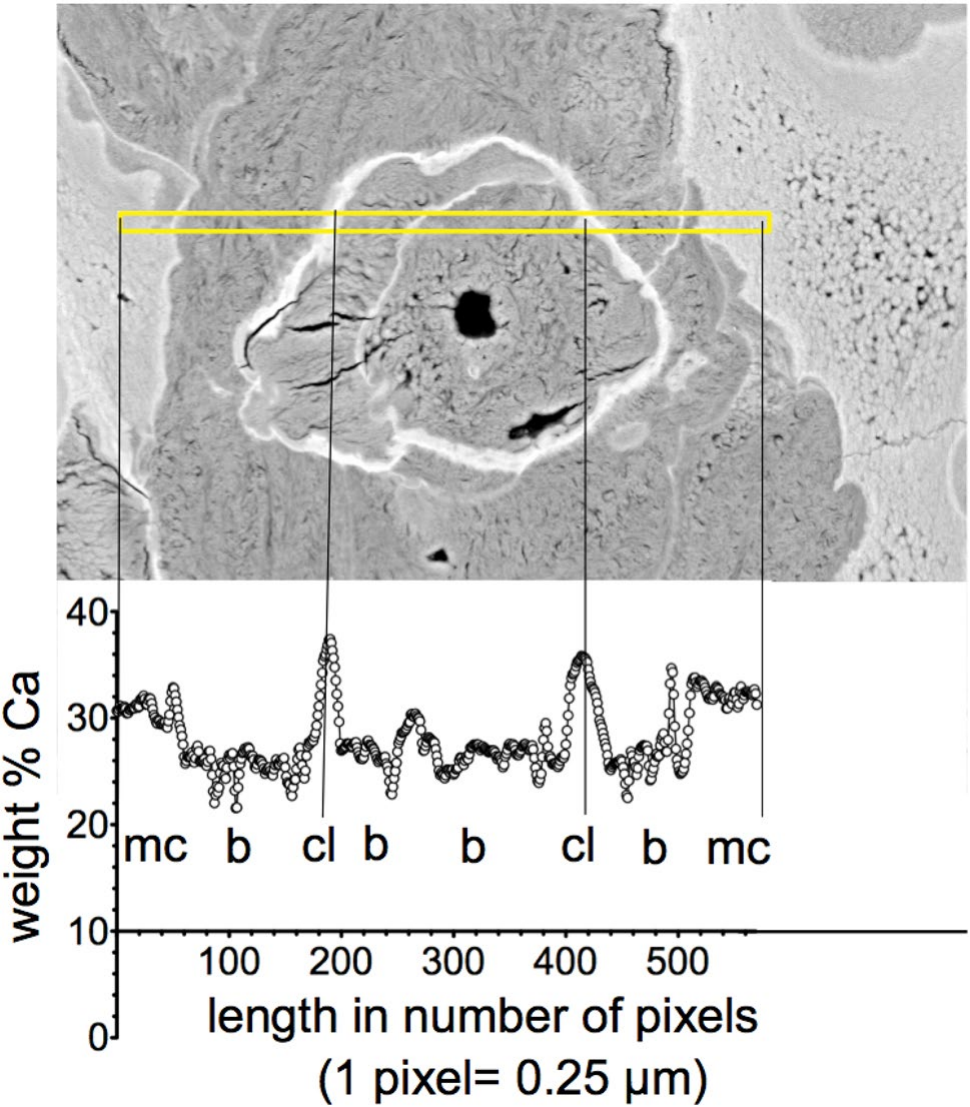
Line profile (yellow indicated line area) of calcium concentration through a tissue area containing mineralized cartilage (mc), bone (b) and thick cement lines (cl)

The extremely high number of cement lines per bone area as visualized by qBEI (Fig. 5) was also noticeable. We could distinguish between two different types of cement lines: thin with normal or increased mineral content and thick with extremely high mineral content (Figs. 5, 6). These highly mineralized cement lines had a thickness up to 15 µm and were unusual in their appearance as they contained frequently small loops and kinks (Figs. 5B–D, 6). A line profile of Ca content through a region of cartilage, bone and cement line clearly demonstrated the extraordinary high mineral content of these cement lines (up to 37 weight% Ca corresponding to 93 weight % hydroxyapatite mineral) (Fig. 6). Moreover, elemental EDX analysis revealed that exclusively in these cement lines, detectable amounts of the element Fluor (0.5 to 1.1 atom %) were present. Furthermore, EDX analysis of the Ca to P ratios showed that the mineral present in mineralized cartilage, bone and cement lines all had a similar calcium-to-phosphorus ratio of 1.71 to 1.76, which is close to the theoretical value of 1.66 of pure hydroxyapatite.

#### OLS

For the OLS characteristics, 1446 osteocyte lacunae sections from the mineralized bone area (mineralized cartilage was excluded for this analysis) were analysed, yielding an OLS porosity of 0.34%, OLS density of 159 per mm^2^, OLS area of 21.27 μm^2^, and OLS perimeter of 19.06 μm, which were all within the range observed in two control samples. However, the proband’s OLS aspect ratio of 2.09 was lower compared to those from the controls (2.62 and 2.54) indicating more roundish osteocyte lacunae.

#### BMDD

The BMDD from our patient was separately measured in the dense region R1 and in the less dense region R2 (see Fig. 5A, E). At both sites, the BMDD was distinctly shifted to higher calcium concentrations and broadened compared to the reference BMDD from healthy adult individuals (Fig. 5E). Consequently, the BMDD parameter values were clearly different from reference data (for BMDD outcomes and *T*-scores from both regions R1 and R2; see Table 2). The average (CaMean *T*-scores + 10.1 and + 6.7 for regions R1 and R2, respectively) and most frequent calcium concentrations (CaPeak *T*-scores + 7.4 and + 5.6), the heterogeneity of mineralization (CaWidth *T*-scores + 4.9 and + 4.4), as well as the percentage of bone with highest mineral content (CaHigh *T*-scores + 19.6 and + 14.6), were highly elevated, while the percentage of low mineralized areas (CaLow *T*-scores − 1.1 and − 0.4) was reduced.

**Table 2:** Comparison of the BMDD-parameters between the osteopetrotic bone biopsy sample and reference data base of healthy adults [19]

BMDD	Reference (*n* = 52)	Patient with osteopetrosis			
		Region R1	R1 *T*-score	Region R2	R2 *T*-score
Ca_MEAN_ [wt% Ca]	22.20 ± 0.45	26.74	+ 10.1	25.21	+ 6.7
Ca_PEAK_ [wt% Ca]	22.94 ± 0.39	25.82	+ 7.4	25.13	+ 5.6
Ca_WIDTH_ [Δwt% Ca]	3.35 ± 0.34	5.03	+ 4.9	4.51	+ 4.4
Ca_LOW_ [%B.Ar]	4.93 ± 1.57	3.18	− 1.1	4.28	− 0.4
Ca_HIGH_ [%B.Ar%]	5.55 ± 3.32	70.65	+ 19.6	54.11	+ 14.6

R1 = dense bone region, R2 = less dense bone region (as indicated in Fig. 5A and E)

Reference data indicate mean ± SD, the patient’s BMDD outcomes are shown additionally by *T*-scores (based on the given reference data)

## Discussion

Here, we describe a patient with profound osteopetrosis, i.e. with extremely elevated bone mass and with vertebrae showing a characteristic “sandwich” structure. The patient had also a history of numerous fractures indicating a high degree in brittleness of the bone material.

Approximately 70% of patients with Albert-Schönberg disease (ADOII) have heterozygous-dominant negative mutations of the *CLCN7* gene (#166600) [6]. In our patient, no amino-acid-converting mutation was evident in the *CLCN7* gene but because of the presence of SNPs in exons 1 and 14, we detected haplo insuffiency in the *CLCN7* gene, with reduced *CLCN7* mRNA expression compared to that of healthy subjects, which likely contributes to the observed phenotype. This did not derive from gene silencing due to promotor methylation. Next generation sequencing revealed the presence of an intronic deletion, insertion and duplication in a homozygous manner, which are not listed or are very rare in the gnomAD and 1000 Genome databases. Furthermore, the intronic duplication introduced new potential SRSF-binding sites, supporting its pathogenic role. All of these variants were homozygous in nature, indicating an unbeknownst distant consanguineous parentage and it is possible that heterozygous occurrence may account for the higher BMD of the proband’s mother, younger sister and nephew, and bone fragility of his nephew; however, this could not be confirmed.

Also, we confirmed that the exonic sequences of all genes known to cause osteopetrosis or high bone mass contained no low-frequency variants that might contribute to the phenotype, with the exception of an amino-acid substitution Arg215Gln in *FERMT3* [25], the gene-encoding Kindlin-3, an integrin-binding protein shown to be important in forming the sealing zone in a bone-resorbing osteoclast [26]. Since arginine to glycine substitutions are frequently responsible for genetic diseases [27], it is possible that this variant contributes to the phenotype of the proband. However, this change was evaluated as “tolerated” by the SIFT [28] and “possibly damaging” by the PolyPhen-2 [29] algorithms.

Reduced transcription of *CLCN7* in this patient might be attributed to the abovementioned homozygous deep intronic duplication, which might alter SRSF-binding sites; however, a precise mechanism for this and the mechanism for the development of CLCN7 haploinsufficiency were not fully elucidated in the current study.

The conclusion from genetic analysis is that the proband phenotype was the result of autosomal recessive inheritance and should, therefore, be classified as ARO rather than ADOII. The homozygous intronic duplication may only cause the suppression of the level of CLCN7, which would explain the relatively mild phenotype comparable to that expected of ARO. Heterozygous inheritance of one or more of these intronic mutations may explain the relatively unremarkable medical histories of the proband’s mother, younger sister and nephew.

In general, defects in the *CLCN7* gene result in reduced bone resorption by osteoclasts and perhaps disturb the coupling between osteoclastic resorption and bone formation by osteoblasts [30]. At an organ level, this dysfunction of osteoclasts leads to the diffuse sclerotic bone phenotype within the entire skeleton, including the characteristic “sandwich vertebrae”, as observed in our patient. In line with the latter, we detected extremely high bone mass at the tissue level and obviously dysfunctional, abnormally shaped giant osteoclasts not necessarily attached to inner bone surfaces, in which large size might be a response to compensate for their defective activity. The mineralized tissue volume was found to be up to 96%, compared to 22% in healthy individuals of the same age [31]. A consequence of the impaired bone (re)modelling in our patient is the persistence of a large fraction of cartilage originating from iliac crest development [32], together with the presence of primary woven bone within mature secondary lamellar bone matrix. Such islands of mineralized cartilage within the bone matrix are a characteristic histologic feature of osteopetrosis [33].

Furthermore, we observed an abnormally high number and increased thickness of cement lines. In general, cement lines are considered to be the first material deposited onto the resorption surface by mononuclear cells [34]. This resorption surface usually shows subtle undulations reflecting the resorption (Howship) lacunae generated by osteoclasts [35]. In our patient, however, numerous cement lines contained frequent small loops and kinks of unknown origin. We hypothesize that these might originate from the inefficient attempts of the osteoclast to resorb the underlying bone material. Moreover, the thickness of the cement lines in our patient was partly up to threefold of the normally observed thickness of 5 microns [35]. In previous histologic assessments of osteopetrotic bone, the presence of an amorphous layer between osteoclasts and the lamellar bone surface was described [33, 36, 37]. Semba et al. [37] reported that this material might contribute to the thickened cement lines in osteopetrosis. In any case, it is well known that, in general, cement lines are more highly mineralized than the surrounding bone matrix [35, 38], which can be attributed to mineralization of non-collagenous protein in the former. Noteworthy, in our patient, most of the cement lines are mineralized to a much higher degree (up to 93 weight% mineral) than usually as can be seen clearly by their brighter appearance in our qBEI images. However, our elemental analysis showed calcium-to-phosphorus ratios consistent with the presence of hydroxyapatite-like mineral in these unusually thick cement lines. Interestingly, we also found an accumulation of fluoride in these cement lines but not in mineralized cartilage or bone. The underlying mechanism and significance of this observation remain unclear.

In addition to affecting osteoclast activity, *CLCN7* deficiency could also affect mineral embedded osteocytes, the predominant cell type in bone [39–41]. We have reported the expression of *CLCN7* in mouse and human osteocytes and have shown that it is upregulated in an in vitro model of perilacunar remodelling [12], a process that reversibly removes mineral from bone, likely for the dual purposes of calcium homeostasis and to maintain an appropriately mineralized bone matrix [39, 42]. The perilacunar and peri-canalicular bone is considered to be a significant calcium reservoir [43, 44], which might be depleted by osteocytic osteolysis in response to high calcium demand [39, 42, 45]. In turn, such a release of calcium would increase the size of the osteocyte lacunae in qBEI images. Low *CLCN7* expression in contrast might hamper the ability of the osteocyte to acidify its surrounding matrix, which is needed during osteocytic osteolysis [12]. Thus, in the proband, the perilacunar and peri-canalicular reservoir is likely highly loaded with calcium, which might contribute to the overall high calcium concentration found in the bone material. In line with this assumption, while we did not observe abnormalities in terms of the number/density or size of osteocyte lacunae, the proband’s bone contained more circular osteocyte lacunae in 2D sections than expected, which may either be attributed to the presence of woven bone [46], or an altered ability of the osteocyte to model its lacunar shape due to a disturbed perilacunar remodelling apparatus. It is noteworthy that several mineralized osteocyte lacunae were also seen in our patient’s bone material reflecting dead osteocytes, which indicate defective bone turnover, repair and impaired bone quality [47].

At the material level, the iliac crest bone biopsy from our patient in general contained greatly increased calcium concentrations and higher heterogeneity of mineralization compared to reference data from healthy adult individuals. This is in line with the high calcium concentrations in CLCN7-dependent osteopetrosis reported by others [48]. At least two factors are contributing to these increased calcium concentrations in our patient, namely the significant amount of mineralized cartilage, which achieves a higher degree of calcium concentration than bone, and increased bone tissue age due to the disturbed bone turnover, thus, leading to prolonged secondary mineralization. At this point, it should be noted that BMDD reflects the history of bone turnover. New bone matrix laid down by osteoblasts onto the cement layer of a resorption lacunae starts to mineralize after a matrix maturation of a few days. The further mineral accumulation occurs in a primary rapid phase (up to about 70% of full mineralization within a few days) and a slower secondary phase (up to full mineralization within months to years) [49, 50]. Thus, bone is composed of bone packets of different ages with correspondingly different mineral contents generating a certain pattern of mineralization as described by the BMDD. In healthy adult individuals, the BMDD in cancellous bone falls within a relatively narrow range, likely reflecting the degree and distribution of mineral to achieve optimal bone strength and toughness [51]. In contrast, the high mineral content in our patient made not only the bone material harder, but also more brittle and prone to fracture [52]. In particular, the deviations from uniform lamellar collagen fibril arrangement in the bone matrix by inclusion of woven bone, cartilage and cement lines contribute to a high heterogeneity of the material introduce weak interfaces, which might act as stress concentrators thus promoting initiation of microcracks [53].

In conclusion, we describe an ARO patient with an ADOII-like phenotype, without an amino-acid-converting mutation but with intronic mutations in *CLCN7* leading to a remarkably increased bone mass. His iliac crest bone consists of lamellar bone but also of inclusion of mineralized cartilage and primary woven bone with abnormally thick and highly mineralized cement lines. These histologic abnormalities are potentially the result of reduced expression of CLCN7 in osteoclasts and possibly osteocytes, causing low rates of bone resorption and the lack of internal bone repair, which is the likely cause of his brittle bone, with reduced fracture resistance.

## Supplementary Information

Below is the link to the electronic supplementary material.

Supplementary file1 (PPTX 384 kb)
