# Supplementary material for: A Mild Case of Autosomal Recessive Osteopetrosis Masquerading as the Dominant Form Involving Homozygous Deep Intronic Variations in the CLCN7 Gene
Source: Calcif Tissue Int. 2022 May 26;111(4):430–44. doi: 10.1007/s00223-022-00988-8 (PMC9474465; doi:10.1007/s00223-022-00988-8)
Supplement: Supplementary file 1 — Supplementary file1 (PPTX 384 kb) [file 223_2022_988_MOESM1_ESM.pptx]

## Slide 1
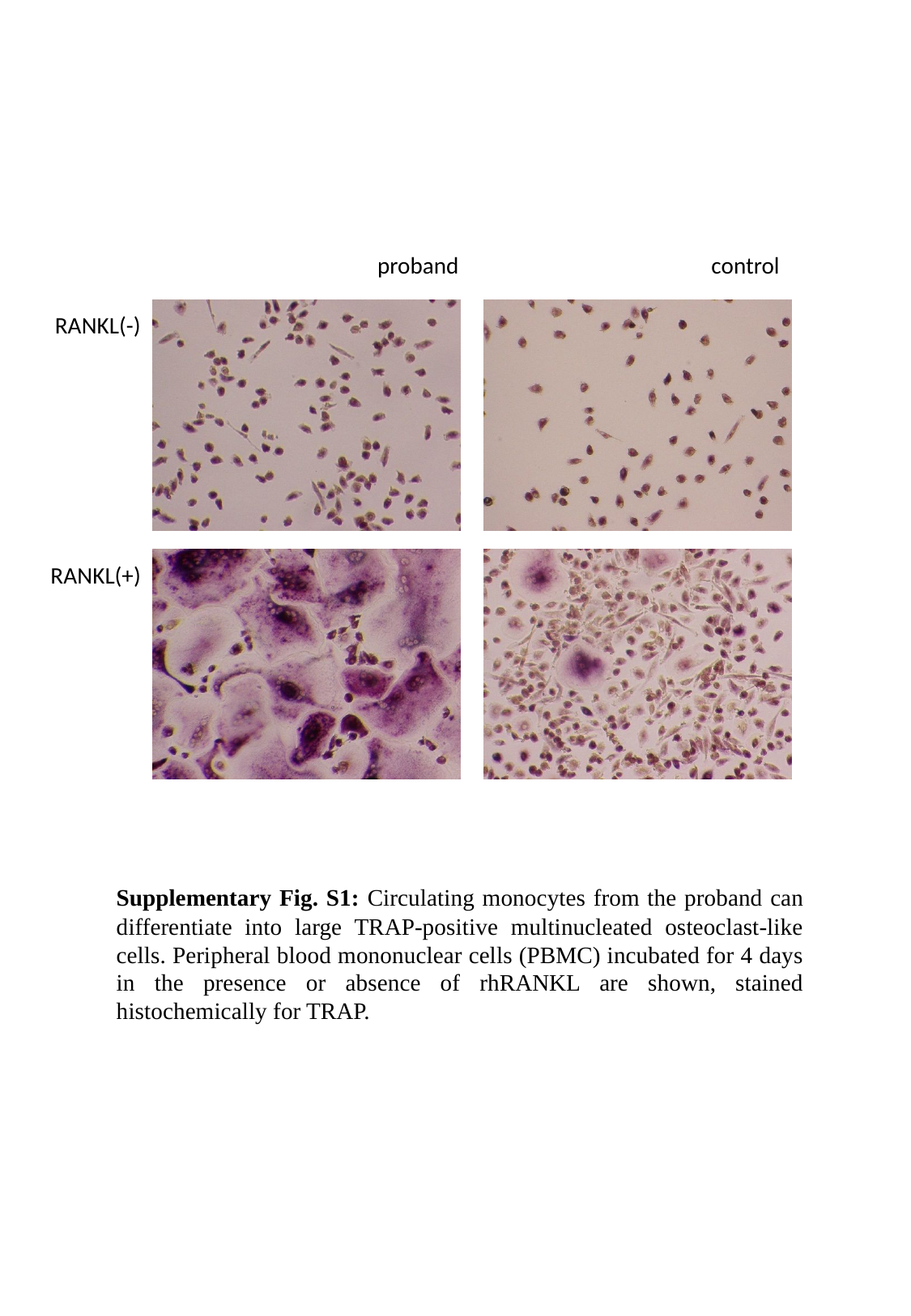

proband
control
RANKL(-)
RANKL(+)
Supplementary Fig. S1: Circulating monocytes from the proband can differentiate into large TRAP-positive multinucleated osteoclast-like cells. Peripheral blood mononuclear cells (PBMC) incubated for 4 days in the presence or absence of rhRANKL are shown, stained histochemically for TRAP.
